# Integration of Curved D-Type Optical Fiber Sensor with Microfluidic Chip

**DOI:** 10.3390/s17010063

**Published:** 2016-12-30

**Authors:** Yung-Shin Sun, Chang-Jyun Li, Jin-Cherng Hsu

**Affiliations:** 1 Department of Physics, Fu-Jen Catholic University, New Taipei City 24205, Taiwan; rmp410797@gmail.com (C.-J.L.); 054326@mail.fju.edu.tw (J.-C.H.); 2 Graduate Institute of Applied Science and Engineering, Fu-Jen Catholic University, New Taipei City 24205, Taiwan

**Keywords:** optical fiber sensor, surface plasmon resonance, biosensor, microfluidic chip

## Abstract

A curved D-type optical fiber sensor (OFS) combined with a microfluidic chip is proposed. This OFS, based on surface plasmon resonance (SPR) of the Kretchmann’s configuration, is applied as a biosensor to measure the concentrations of different bio-liquids such as ethanol, methanol, and glucose solutions. The SPR phenomenon is attained by using the optical fiber to guide the light source to reach the side-polished, gold-coated region. Integrating this OFS with a polymethylmethacrylate (PMMA)-based microfluidic chip, the SPR spectra for liquids with different refractive indices are recorded. Experimentally, the sensitivity of the current biosensor was calculated to be in the order of 10^−5^ RIU. This microfluidic chip-integrated OFS could be valuable for monitoring subtle changes in biological samples such as blood sugar, allergen, and biomolecular interactions.

## 1. Introduction

The surface plasmon resonance (SPR) phenomenon occurs as the consequence of exciting a surface-bound electromagnetic wave at the interface between a metal and a transparent material. Biosensors based on this technique have been designed and constructed for label-free and real-time detection of biomolecular interactions [1,2,3,4,5,6]. In 2007, 96% of biosensing-related publications were based on SPR-related technologies [7]. To attain the SPR phenomenon, the Kretchmann’s configuration or the surface-grating modification is used [8]. In the Kretchmann’s configuration, a glass prism is usually required to achieve total internal reflection (TIR) [8], and such configuration is applied in both SPR spectroscopy and SPR microscopy [9,10,11,12,13]. As an alternative, the grating-coupled SPR takes place when the light diffracted by the grating has different orders with at least one inducing SPR [14,15,16]. In these two configurations, bulk optical components and/or complicated surface modifications are required, preventing them from in vivo applications. As a result, optical fibers, characterized by their small size, low cost, and flexibility, provide a TIR configuration for attaining the SPR phenomenon. Various types of SPR-based optical fiber sensors (OFSs) have been proposed for measuring changes in refractive index, pressure, and temperature [17,18], as well as for detecting biomaterials and biomolecules [19,20,21]. One of the very first SPR-based OFSs was reported by Villuendas and Pelayo in 1990 [22]. The excitation and detection conditions were extensively analyzed in three near-IR wavelengths of 0.85 μm, 1.3 μm, and 1.55 μm. The sensitivity and dynamic range of this OFS in detecting aqueous sucrose solutions were determined to be 3.5 × 10^−6^ RIU and be 3.5 × 10^−3^ RIU, respectively, in the 1.55 μm wavelength. Bardin et al. applied an optical fiber with an inverted graded-index (IGI) profile in measuring changes of the transmitted power caused by refractive-index variations of the surrounding dielectric medium [23]. A resolution of 5 × 10^−5^ RIU was achieved in a range of the refractive index of the tested medium between 1.33 and 1.39. Hautakorpi et al. proposed a novel SPR-based OFS based on coating the holes of a three-hole microstructured optical fiber with a low-index dielectric layer [24]. The results indicated that the optical loss of the Gaussian guided mode could be made very small by tuning the thickness of the dielectric layer and that the refractive-index resolution for aqueous analytes is 1 × 10^−4^ RIU.

D-shaped and D-type OFSs have also been reported for their easy fabrication and high sensitivity. In a D-shaped OFS, the cladding is partially polished, while in a D-type OFS, the core is side-polished. Lo et al. proposed a four-layer D-shaped fiber SPR sensor for strain sensing applications, where the phase difference between the *p*- and *s*-polarized waves is measured using a common-path heterodyne interferometer [25]. The experimental results have shown that the sensitivity of this OFS (around 2.19 × 10^4^ deg/ε) is significantly higher than that of a conventional polarimetric fiber sensor (around 5.2 × 10^2^ deg/ε). Luan et al. presented a D-shaped hollow core microstructured optical fiber (MOF)-based SPR sensor, where the air hole in the fiber core can lower the refractive index of a Gaussian-like core mode to match with that of a plasmon mode [26]. A D-type OFS with a thin gold film was reported by Wang et al. to have a sensitivity of 8 × 10^−5^ RIU [27]. By optimizing parameters of the OFS, such as the fiber size, the coating thickness, the wavelength, and the incident angle, the sensitivity could be enhanced to 2.5 × 10^−5^ RIU [28]. In 1996, Homola and Slavik reported the first curved D-type OFS with a sensitivity of 2 × 10^−5^ RIU in the refractive index range of 1.41–1.42 [29]. These curved fiber sensors were considered to be more sensitive and more stable than other types of OFSs [18], yet due to the nature of the curvature, the simulation and fabrication of these curved devices were much more complicated than others. Recently, Hsu et al. studied the curved D-type OFSs in detail by calculating the number of reflections within the fiber sensing region and simulating the reflectance at combined parameters of gold thickness of the deposited film, the incident angle, and the refractive index of ambient [30]. Experiments were also performed to monitor the SPR phenomena of ethylene glycol solutions with different refractive indexes. Sensitivities of 2.03 × 10^−5^ RIU and 2.05 × 10^−4^ RIU were derived theoretically and experimentally, respectively, with a wide dynamic range of 1.33–1.43.

For liquid sensing, the sensing region of the OFS is covered with drops of liquids whose refractive indices are to be determined. However, being a biosensor for detecting biomolecular interactions, the performance of the OFS is limited by the number of analytes present near the sensing region. In situations where analytes have to diffuse and propagate through the bulk solution to reach the sensing region, the mass-transport effect could affect the detection limit, response time, and kinetic analysis of the sensor [31,32,33]. This mass-transport effect could, to some extent, be overcome by continuously flowing fresh solutions across the sensing surface, which is realized by combing the biosensor with the microfluidic device. Microfluidic devices provide a miniaturized platform with advantages of low reagent consumption, shortened processing time, low cost, and easy fabrication. For example, Hsu et al. reported the integration of the fiber optic-particle plasmon resonance (FO-PPR) biosensor with a polymethylmethacrylate (PMMA)-based microfluidic channel of 4.0 cm × 900 μm × 900 μm to reduce response time and improve detection limit [34]. The resolution of the refractive index of various sucrose solutions was enhanced by 2.4-fold in the microfluidic system. Makiabadi et al. also integrated an optical fiber SPR sensor with a microfluidic system to monitor in real-time the sensitivity of OFS for each kinetic reaction occurring at the surface [35]. In this paper, we combined a curved D-type optical fiber sensor with a microfluidic chip for sensing bio-liquids with different refractive indices. This work corresponds to the experimental part of the reference [30]. Experiments were performed to attain a sensitivity of 10^−5^ RIU. The results suggest that this microfluidic chip-integrated OFS can serve as a biosensor for sensitive, label-free, real-time, and in-situ monitoring of biomolecular interactions.

## 2. Materials and Methods

### 2.1. SPR Theory

**Figure 1:**
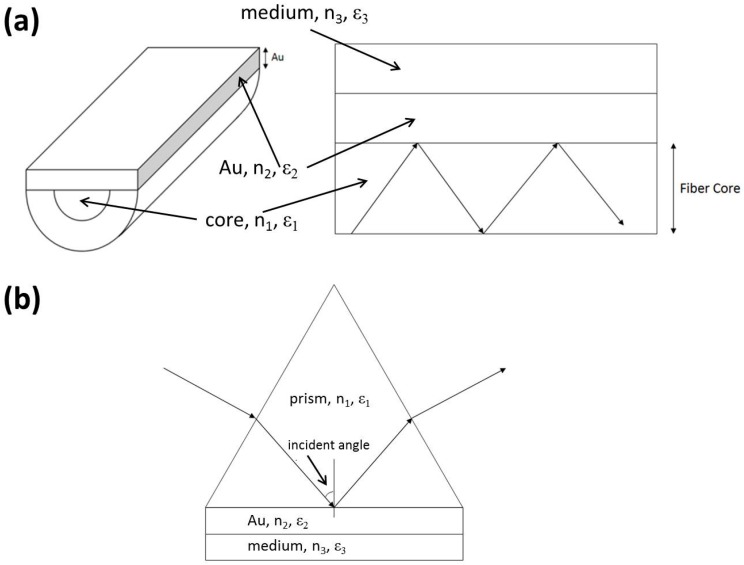
The schemes of (**a**) the optical fiber sensor and (**b**) the Kretchmann’s configuration.

As shown in Figure 1a, the core of a D-type OFS is side-polished and coated with a thin gold film to achieve the SPR phenomenon. Figure 1b shows the three-layered Kretchmann’s configuration, where an evanescent wave propagates along the metal-dielectric interface. This evanescent wave can interact with the plasma waves on the surface, excite the plasmons, and then cause resonance. Detailed calculation of the SPR theory was presented in the reference [30].

### 2.2. Simulation

The simulation includes two parts: (1) Calculating the number of reflections and the incident angles under different fiber curvature and core-polished depth; (2) Using an optical thin-film software (the Essential Macleod software package from the Thin Film Center Inc., Tucson, AZ, USA) to simulate the dependence of reflectance on the fiber curvature, the core-polished depth, and the gold-film thickness. The simulation results, showing a sensitivity of around 2 × 10^−5^ to 3 × 10^−5^ RIU, were detailed in the reference [30].

### 2.3. Fabrication of the OFS

**Figure 2:**
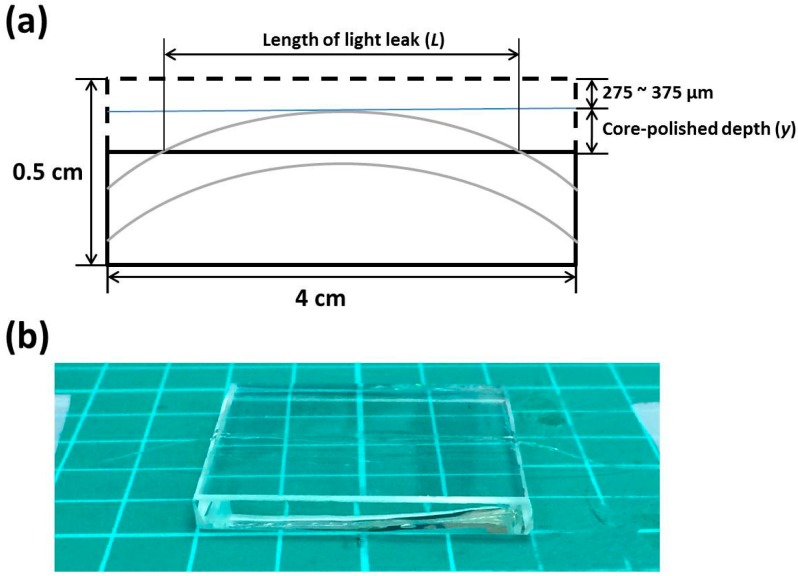
The (**a**) side-view scheme and (**b**) picture of the embedded curved optical fiber.

Multi-mode optical fibers with a core diameter of 62.5 μm, a cladding diameter of 125 μm, and a numerical aperture (NA) of 0.272 were used in this study, providing higher “light-gathering” capacity than single-mode fibers. First, a trench with a curvature of 580 mm was created, using a trench-cutting machine, on a 4 cm × 4 cm × 0.5 cm glass substrate. As shown in Figure 2a, a thickness of 275 μm to 375 μm was left on the top of the glass slide for polishing. Then an optical fiber was pushed against the trench and embedded inside by filling the trench with a UV-curing adhesive. Figure 2b shows a picture of the embedded curved optical fiber. This curvature-trench method has the following advantages: (1) The glass substrate with a large surface area can reduce the pressure and prevent the fiber from breaking during the polishing process; (2) the curvature of the fiber can be precisely controlled for calculating the core-polished depth. 

Next, a grinding machine was used to polish the optical fiber-embedded glass slide. The grinding powders with different grain sizes, from ~11.3 μm of Al_2_O_3_ powder #800, ~7.1 μm of Al_2_O_3_ powders #1200, to <0.3 μm of CeO_2_ burnishing powders, were mixed with water, step-by-step, for different polishing process. A red semiconductor laser of 633 nm was induced to the fiber. As the fiber core was partially polished, a leak of light occurred in the desired sensing region. As shown in Figure 2b, the core-polished depth (*y*) can be related to the curvature of the fiber (*R* = 580 mm) and the length of the light leak (*L*) as

(1) $$y=R-\sqrt{R^{2}-{\left(\frac{L}{2}\right)}^{2}}$$

By coupling the red semiconductor laser beam into the fiber, the length of the light leak was measured to be around 1.1–1.25 cm. This resulted in a core-polished depth to be about 26–33.7 μm.

Finally, a thin gold film was deposited on the fiber-embedded glass substrate. Before sputtering, the chamber was vacuumed to a background pressure of 2 × 10^−5^ Torr. During sputtering, Ar gas at a rate of 14 sccm was flowed into the chamber to attain a working pressure of 2 × 10^−3^ Torr. With a DC sputtering power of 60 W, the thickness of the deposited film around 10–12 nm was in-situ monitored with a quartz crystal thickness monitor and ex-site checked with an ellipsometer (VASE M-2000U, J. A. Woollam Company, Lincoln, NE, USA).

### 2.4. Fabrication of the Microfluidic Chip

**Figure 3:**
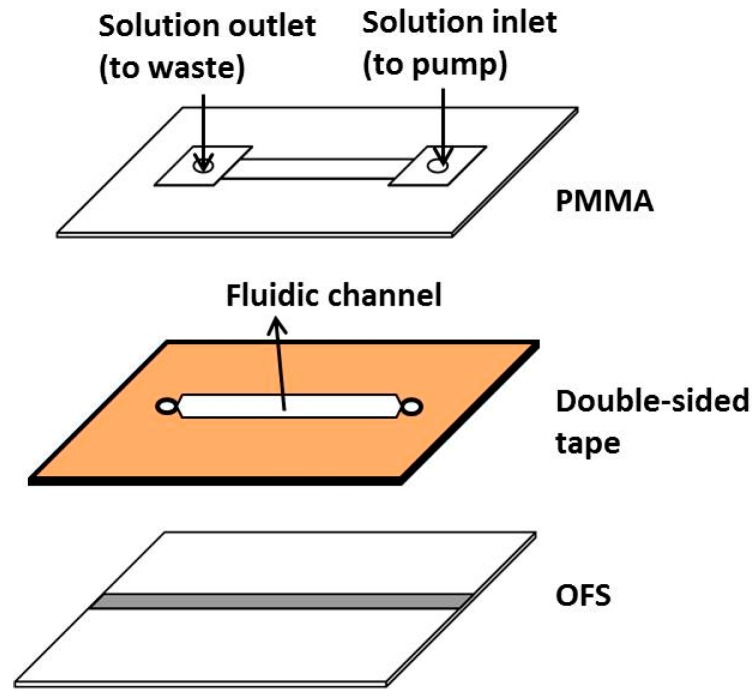
The design of the microfluidic chip composed of a polymethylmethacrylate (PMMA) substrate, a double-sided tape, and a D-type optical fiber sensor (OFS) embedded inside a glass substrate.

The design of the microfluidic chip is shown in Figure 3. The pattern was drawn in AutoCAD (version 2014, Autodesk , San Rafael, CA, USA) and then loaded into a CO_2_ laser scriber (ILS2, Laser Tools & Technics Corp., Hsinchu City, Taiwan) to ablate the desired patterns on polymethylmethacrylate (PMMA) substrates and double-sided tapes (8018, 3M, St. Paul, MN, USA) [36,37,38,39]. The top PMMA layer had two holes for solution inlet and outlet. The middle double-sided tape, having a channel size of 2 cm × 0.5 cm × 26 μm, served as the flow cell. This tape bound to the bottom OFS to form the integrated device.

### 2.5. Materials

Bio-liquids with different refractive indices were prepared and flowed across the sensing region of the OFS for observing the SPR spectra. Four sets of liquids were made, and their refractive indices were calculated and measured using an Abbe refractometer.

1. For ethanol, the following equation is applied:
  (2) $$n_{\mathrm{mix}}=n_{\mathrm{water}}+0.0522\;\times \;\left(\frac{V_{\mathrm{eth}}}{V_{\mathrm{tot}}}\right)$$
  In this equation [40,41], *n*_mix_ and *n*_water_ are the refractive indices of ethanol-water mixture and water, respectively, and *V*_eth_/*V*_tot_ is the volume ratio of ethanol to total solution. Assuming *n*_water_ = 1.33, refractive indices of 1.3412, 1.3429, 1.3456, 1.3499, and 1.3587 were obtained by suitably adjusting *V*_eth_/*V*_tot_. These values were further measured to be 1.3411, 1.3427, 1.346, 1.3507, and 1.358.
2. For methanol, the following equation is applied:
  (3) $$n_{\mathrm{mix}}=n_{\mathrm{water}}+0.01738\;\times \;\left(\frac{V_{\mathrm{meth}}}{V_{\mathrm{tot}}}\right).$$
  Using this equation, refractive indices of 1.3354, 1.336, 1.3368, 1.3383, and 1.3412 were obtained by suitably adjusting *V*_meth_/*V*_tot_. These values were further measured to be 1.335, 1.3362, 1.3375, 1.3385, and 1.3402.
3. For ethanol-methanol mixture, the following equation is applied:
  (4) $$n_{\mathrm{mix}}=n_{\mathrm{water}}+0.01738\;\times \;\left(\frac{V_{\mathrm{meth}}}{V_{\mathrm{tot}}}\right)+0.0522\;\times \;\left(\frac{V_{\mathrm{eth}}}{V_{\mathrm{tot}}}\right).$$
  Using this equation, refractive indices of 1.3412, 1.3552, 1.3569, 1.3578, and 1.3587 were obtained by suitably adjusting *V*_meth_/*V*_tot_ and *V*_eth_/*V*_tot_. These values were further measured to be 1.3402, 1.3547, 1.3553, 1.3563, and 1.358.
4. For glucose solution, the following equation is applied:
  (5) $$n_{\mathrm{mix}}=n_{\mathrm{water}}+0.101567\;\times \;\left(\frac{M_{\mathrm{glu}}}{V_{\mathrm{tot}}}\right).$$
  In this equation, *M*_glu_/*V*_tot_ is the mass concentration (g/mL) of the solution. By suitably adjusting *M*_glu_/*V*_tot_, refractive indices of 1.343, 1.353, 1.363, 1.3731, and 1.383 were obtained. These values were further measured to be 1.3431, 1.3531, 1.3635, 1.3735, and 1.379.

### 2.6. Experimental Setup

**Figure 4:**
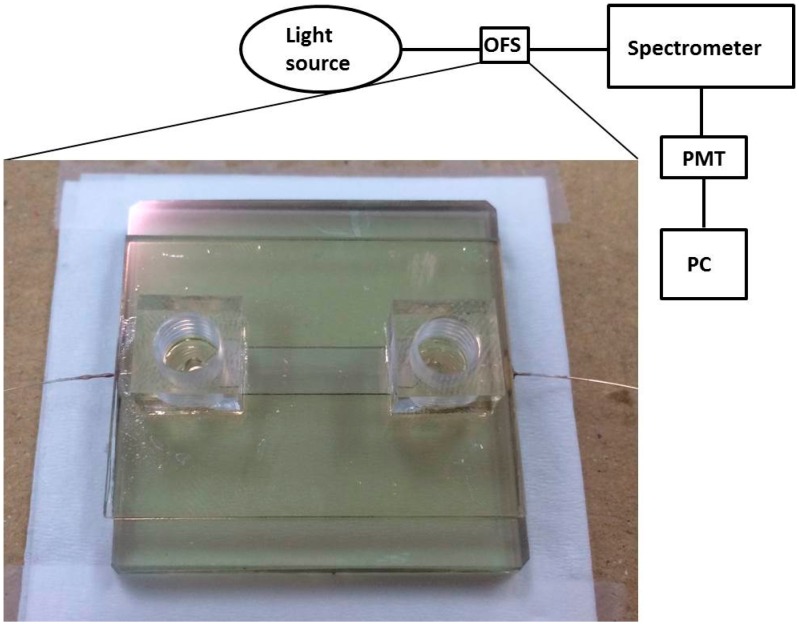
The experimental setup composed of a light source, the microfluidic chip-integrated OFS, a spectrometer, and a photomultiplier (PMT).

As shown in Figure 4, the experimental setup contained a light source, the microfluidic chip-integrated OFS, a photoluminescence (PL) spectrometer, and a photomultiplier (PMT). A tungsten halogen lamp (LS-1, Ocean Optics, Dunedin, FL, USA) with a spectrum between 400 nm and 900 nm was used as the unpolarized light source. This light source was directly coupled into the optical fiber at incident angles between 80° and 90° in the polished region, and the output light was measured with the PL spectrometer (Triax 320, HORIBA, Kyoto, Japan). The integration time was set to be 0.1 s, and the scanning wavelength was set to range from 400 nm to 900 nm with a resolution of 0.06 nm. Finally, a PMT (R5108, Hamamatsu Photonics, Tokyo, Japan), having a detection range of 400 nm–1200 nm and the strongest response at 800 nm, converted the light signals to electronic signals before they were further processed by a computer [30]. The bio-liquid to be analyzed was continuously flowed across the sensing region of the OFS using a syringe pump (NE-300, New Era, Farmingdale, NY, USA) at a flow rate of 40 μL/min. A stable SPR spectrum was recorded after about 10 min of waiting. For statistical analysis, four SPR spectra were collected for one bio-liquid (twice from large *n* to small *n*, and twice the reversed way).

## 3. Results

### 3.1. The SPR Spectra

**Figure 5:**
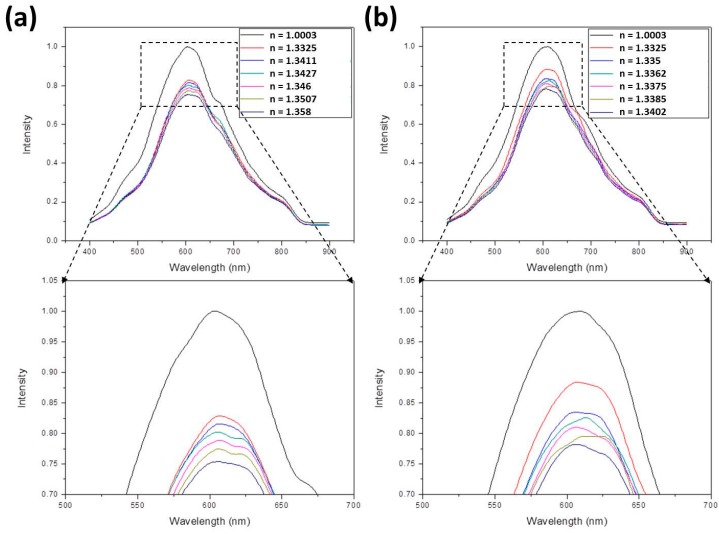
The normalized surface plasmon resonance (SPR) spectra of (**a**) ethanol and (**b**) methanol solutions with different refractive indices.

**Figure 6:**
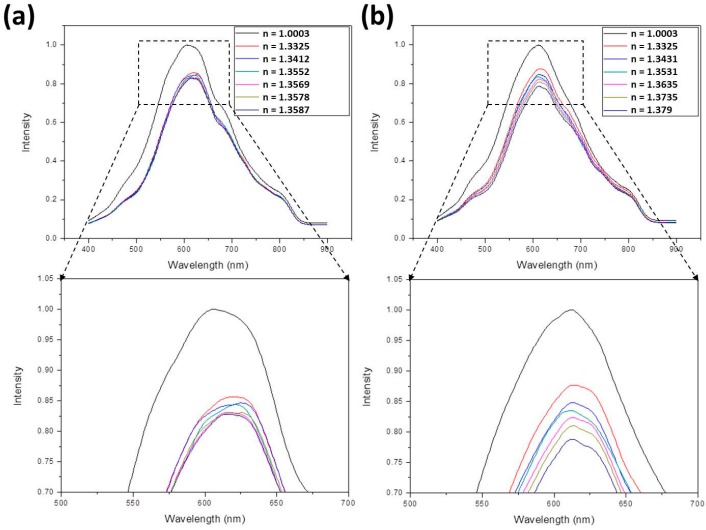
The normalized SPR spectra of (**a**) ethanol-methanol and (**b**) glucose solutions with different refractive indices.

Figure 5a shows the normalized SPR spectra of ethanol solutions with different refractive indices, which were measured by the Abbe refractometer, from small *n* (*n*_air_ = 1.0003) to large *n* (*n* = 1.358 for *V*_eth_/*V*_tot_ = 1). The enlarged figure indicates the maximum intensities, being 1, 0.829, 0.816, 0.802, 0.788, 0.774, and 0.754 for *n* = 1.003, 1.3325, 1.3411, 1.3427, 1.346, 1.3507, and 1.358, respectively (all refractive indices were measured values). These peak values decreased with increasing refractive index. Figure 5b shows the normalized SPR spectra of methanol solutions with different refractive indices from small *n* (*n*_air_ = 1.0003) to large *n* (*n* = 1.3402 for *V*_meth_/*V*_tot_ = 1). As indicated in the enlarged figure, the maximum intensities decreased with increasing refractive index, being 1, 0.884, 0.835, 0.825, 0.81, 0.795, and 0.782 for *n* = 1.003, 1.3325, 1.335, 1.3362, 1.3375, 1.3385, and 1.3402, respectively (all refractive indices were measured values). Figure 6a shows the normalized SPR spectra of ethanol-methanol solutions with different refractive indices from small *n* (*n*_air_ = 1.0003) to large *n* (*n* = 1.358 for *V*_eth_/*V*_tot_ = 1 and *V*_meth_/*V*_tot_ = 0). The enlarged figure indicates decreased maximum intensities with increasing refractive index, being 1, 0.856, 0.847, 0.844, 0.831, 0.83, and 0.828 for *n* = 1.003, 1.3325, 1.3402, 1.3547, 1.3553, 1.3563, and 1.358, respectively (all refractive indices were measured values). Figure 6b shows the normalized SPR spectra of glucose solutions with different refractive indices from small *n* (*n*_air_ = 1.0003) to large *n* (*n* = 1.379 for *M*_glu_/*V*_tot_ = 0.5). As indicated in the enlarged figure, the maximum intensities decreased with increasing refractive index, being 1, 0.877, 0.849, 0.835, 0.824, 0.81, and 0.788 for *n* = 1.003, 1.3325, 1.3431, 1.3531, 1.3635, 1.3735, and 1.379, respectively (all refractive indices were measured values).

### 3.2. The Sensitivity

**Figure 7:**
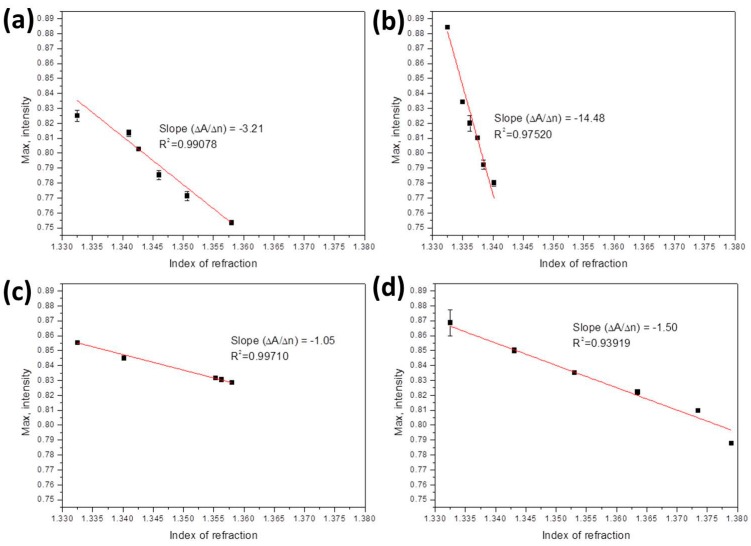
The maximum intensities as a function of the refractive index for (**a**) ethanol solutions; (**b**) methanol solutions; (**c**) ethanol-methanol solutions; and (**d**) glucose solutions. In each panel, the slope Δ*A*/Δ*n* was derived by linearly fitting all points.

The sensitivity *S* (in RIU) of the OFS can be defined as [27]

(6) $$S=\frac{∆T}{\left(\frac{∆A/N}{∆n}\right)}$$

where Δ*T* is the resolution in the intensity of the light source, *N* is the normalization factor, and Δ*A*/Δ*n* is the slope of the maximum intensity to the refractive index. In Figure 5 and Figure 6, all SPR spectra are normalized to the peak value of *n*_air_. Thus, the value of $\left(\frac{\frac{\Delta A}{N}}{\Delta n}\right)$ can be derived by plotting the normalized maximum intensity with respect to the refractive index, as shown in Figure 7. In each panel, all points are linearly fitted to obtain a negative slope due to the decreasing maximum intensities with increasing refractive index. With a light source resolution of 0.01%, the sensitivity was calculated by using Equation (6). Table 1 lists the slopes and sensitivities for different bio-liquids.

## 4. Discussion

For ethanol sensing, the sensitivity was calculated to be 3.12 × 10^−5^ RIU. Using Equation (2), this corresponded to a minimum detectable *V*_eth_/*V*_tot_ of 6 × 10^−4^, 0.06%, or 60 ppm. This sensitivity was comparable to those observed in other OFSs. For example, Song et al. developed a highly sensitive ethanol sensor based on mesoporous ZnO–SnO_2_ nanofibers, and it was claimed that this sensor had advantages of high sensitivity, quick response and recovery, good reproducibility, and linearity in the range of 3–500 ppm [42]. Shabaneh et al. reported using a multi-mode, carbon nanotubes (CNT)-coated fiber to detect ethanol solutions with concentration ranges of 5%–80%, and found that the sensor reflectance reduced proportionally [43]. To detect the alcohol concentration in liquors, Morisawa and Muto fabricated an OFS where a mixture polymer of novolac resin and polyvinylidenefluoride (PVDF) was coated as a sensitive cladding layer on the plastic fiber core [44]. Using this sensor, optical detection of the alcohol concentration in real liquors, for example, from beer (5 *v*/*v*%) to whisky (40 *v*/*v*%) could easily be obtained with a fast response time less than one minute. 

For methanol sensing, the sensitivity was calculated to be 6.91 × 10^−6^ RIU. Using Equation (3), this corresponded to a minimum detectable *V*_meth_/*V*_tot_ of 4 × 10^−4^, 0.04%, or 40 ppm. Usually, methanol sensors were constructed based on electrochemical techniques. Rahman et al. deposited calcined α-Fe_2_O_3_ co-doped SnO_2_ nanocubes (NCs) on a silver electrode (AgE) to give a sensor with a fast response towards methanol in liquid phase [45]. This sensor exhibited good sensitivity and long-term stability, and enhanced electrochemical response, with a detection limit of 0.16 mmol∙L^−1^ (about 6.5 ppm). The same group also fabricated a methanol sensor by depositing silver oxide nanoparticles (NPs) on a glassy carbon electrode [46]. The detection limit was claimed to be 36.0 μM (around 1.5 ppm). Compared with these electrochemical methods, the current OFS provides advantages of small size, low cost, flexibility, and easy fabrication, despite a slightly worse sensitivity.

For methanol-ethanol mixture sensing, the sensitivity was calculated to be 9.52 × 10^−5^ RIU. Using Equation (4), this corresponded to a minimum detection limit of 2.73 × 10^−3^, 0.273%, or 273 ppm, if ethanol was replaced by methanol. This OFS could be helpful to distinguishing adulterated from authorized wines. Based on deformed optical fiber cores, Fabian et al. reported an optical fiber evanescent field sensor for methanol and ethanol solvents [47]. Using the visible wavelength at 650 nm, it was shown that the sensitivity of this sensor enabled the determination of methanol concentrations of better than 0.5% and of ethanol concentrations of better than 0.2%.

For glucose sensing, the sensitivity was calculated to be 6.67 × 10^−5^ RIU. Using Equation (5), this corresponded to a minimum detectable *M*_glu_/*V*_tot_ of 66 mg/dL. This OFS could be helpful to monitoring the level of bold sugar, and its sensitivity was comparable to those of other sensors. For example, Wang et al. described a fiber loop ring-down glucose sensor using refractive index-difference evanescent field attenuation effect as the sensing mechanism [48]. The estimated theoretical detection sensitivity of this glucose sensor was 10 mg/dL, which was approximately 17 times lower than the glucose renal threshold concentration. Luo et al. reported a highly sensitive, label-free and selective glucose sensor by using excessively tilted fiber grating (Ex-TFG) inscribed in the thin-cladding optical fiber (TCOF) [49]. The experimental results showed that Ex-TFG in TCOF based sensor had a reliable and fast detection of the glucose concentration as low as 10 mg/dL. Li and Walt developed a fiber-optic glucose-oxygen sensor comprised of dual-analyte sensing sites in the defined positions on the distal end of an imaging fiber [50]. The glucose detection limit was shown to be around 0.6 mM (1.08 mg/dL).

## 5. Conclusions

In this paper, the combination of a curved D-type optical fiber sensor with a microfluidic chip was reported. This OFS was used as a biosensor to measure the refractive indices of different bio-liquids such as ethanol, methanol, and glucose solutions. Its sensitivity was around 10^−5^ RIU, and this value was comparable to those derived in other ethanol or glucose sensors. By virtue of the advantages of this microfluidic chip-integrated OFS, e.g., small size, low cost, flexibility, easy fabrication, low sample consumption, and in vivo measurement, this device could have great applications as a biosensor for monitoring subtle changes in biological samples such as blood sugar, allergen, and biomolecular interactions.

## Figures and Tables

**Table 1:** The slopes and sensitivities for different bio-liquids.

Bio-Liquids	Refractive Index	Slope	Sensitivity	Previous Report Sensitivity
**Ethanol solutions**	1.3325–1.358	–3.21	3.12 × 10^−5^ RIU or 0.06% or 60 ppm	3–500 ppm [42]; 5%–80% [43]; 5%–40% [44]
**Methanol solutions**	1.3325–1.3402	–14.48	6.91 × 10^−6^ RIU or 0.04% or 40 ppm	6.5 ppm [45]; 1.5 ppm [46]
**Ethanol-methanol**	1.3325–1.358	–1.05	9.52 × 10^−5^ RIU or 0.273%/273 ppm of methanol	Methanol 0.5% [47]
**Glucose solutions**	1.3325–1.379	–1.5	6.67 × 10^−5^ RIU or 66 mg/dL of glucose	10 mg/dL [48,49]; 1.08 mg/dL [50]

## References

[1] Pilolli R., Visconti A., Monaci L. (2015). Rapid and label-free detection of egg allergen traces in wines by surface plasmon resonance biosensor. Anal. Bioanal. Chem..

[2] Li Y.H., Yan Y.R., Lei Y.N., Zhao D., Yuan T.X., Zhang D.C., Cheng W., Ding S.J. (2014). Surface plasmon resonance biosensor for label-free and highly sensitive detection of point mutation using polymerization extension reaction. Colloid Surf. B.

[3] Zhang D.C., Yan Y.R., Li Q., Yu T.X., Cheng W., Wang L., Ju H.X., Ding S.J. (2012). Label-free and high-sensitive detection of Salmonella using a surface plasmon resonance DNA-based biosensor. J. Biotechnol..

[4] Hu J.D., Li W., Wang T.T., Lin Z.L., Jiang M., Hu F.J. (2012). Development of a label-free and innovative approach based on surface plasmon resonance biosensor for on-site detection of infectious bursal disease virus (IBDV). Biosens. Bioelectron..

[5] Huang C.J., Bonroy K., Reekman G., Verstreken K., Lagae L., Borghs G. (2009). An on-chip localized surface plasmon resonance-based biosensor for label-free monitoring of antigen-antibody reaction. Microelectron. Eng..

[6] Huang H., He C.C., Zeng Y.L., Xia X.D., Yu X.Y., Yi P.G., Chen Z. (2009). A novel label-free multi-throughput optical biosensor based on localized surface plasmon resonance. Biosens. Bioelectron..

[7] Rich R.L., Myszka D.G. (2008). Survey of the year 2007 commercial optical biosensor literature. J. Mol. Recognit..

[8] Sun Y.S. (2014). Optical Biosensors for Label-Free Detection of Biomolecular Interactions. Instrum. Sci. Technol..

[9] Kurihara K., Suzuki K. (2002). Theoretical understanding of an absorption-based surface plasmon resonance sensor based on Kretchmann’s theory. Anal. Chem..

[10] Keske A., Atar A., Ustundag I., Caglayan M.O. (2014). Detection of Influenza A by Surface Plasmon Resonance Enhanced Total Internal Reflection Ellipsometry. J. Comput. Theor. Nanosci..

[11] Ustundag Z., Caglayan M.O., Guzel R., Piskin E., Solak A.O. (2011). A novel surface plasmon resonance enhanced total internal reflection ellipsometric application: Electrochemically grafted isophthalic acid nanofilm on gold surface. Analyst.

[12] Abbas A., Linman M.J., Cheng Q. (2011). New trends in instrumental design for surface plasmon resonance-based biosensors. Biosens. Bioelectron..

[13] Scarano S., Mascini M., Turner A.P., Minunni M. (2010). Surface plasmon resonance imaging for affinity-based biosensors. Biosens. Bioelectron..

[14] Bahrami F., Aitchison J.S., Mojahedi M. (2015). Dual-Wavelength Spectroscopy of a Metallic-Grating-Coupled Surface Plasmon Resonance Biosensor. IEEE Photonics J..

[15] Marusov G., Sweatt A., Pietrosimone K., Benson D., Geary S.J., Silbart L.K., Challa S., Lagoy J., Lawrence D.A., Lynes M.A. (2012). A Microarray Biosensor for Multiplexed Detection of Microbes Using Grating-Coupled Surface Plasmon Resonance Imaging. Environ. Sci. Technol..

[16] Wang Y., Dostalek J., Knoll W. (2011). Magnetic Nanoparticle-Enhanced Biosensor Based on Grating-Coupled Surface Plasmon Resonance. Anal. Chem..

[17] Sharma A.K., Gupta B.D. (2006). Theoretical model of a fiber optic remote sensor based on surface plasmon resonance for temperature detection. Opt. Fiber. Technol..

[18] Sharma A.K., Jha R., Gupta B.D. (2007). Fiber-optic sensors based on surface plasmon resonance: A comprehensive review. IEEE Sens. J..

[19] Sciacca B., Monro T.M. (2014). Dip biosensor based on localized surface plasmon resonance at the tip of an optical fiber. Langmuir.

[20] Sai V.V., Kundu T., Mukherji S. (2009). Novel U-bent fiber optic probe for localized surface plasmon resonance based biosensor. Biosens. Bioelectron..

[21] Lin T.J., Chung M.F. (2009). Detection of cadmium by a fiber-optic biosensor based on localized surface plasmon resonance. Biosens. Bioelectron..

[22] Villuendas F., Pelayo J. (1990). Optical fibre device for chemical seming based on surface plasmon excitridon. Sens. Actuators A.

[23] Bardin F., Ivan K., Trouillet A., Matejec V., Gagnaire H., Chomat M. (2002). Surface plasmon resonance sensor using an optical fiber with an inverted graded-index profile. Appl. Opt..

[24] Hautakorpi M., Mattinen M., Ludvigsen H. (2008). Surface-plasmon-resonance sensor based on three-hole microstructured optical fiber. Opt. Express.

[25] Lo Y.L., Chuang C.H., Lin Z.W. (2011). Ultrahigh sensitivity polarimetric strain sensor based upon D-shaped optical fiber and surface plasmon resonance technology. Opt. Lett..

[26] Luan N., Wang R., Lv W., Yao J. (2015). Surface plasmon resonance sensor based on D-shaped microstructured optical fiber with hollow core. Opt. Express.

[27] Wang S.F., Chiu M.H., Hsu J.C., Chang R.S., Wang F.T. (2005). Theoretical analysis and experimental evaluation of D-type optical fiber sensor with a thin gold film. Opt. Commun..

[28] Chiu M.H., Shih C.H., Chi M.H. (2007). Optimum sensitivity of single-mode D-type optical fiber sensor in the intensity measurement. Sens. Actuators B-Chem..

[29] Homola J., Slavik R. (1996). Fibre-optic sensor based on surface plasmon resonance. Electron. Lett..

[30] Hsu J.C., Jeng S.W., Sun Y.S. (2015). Simulation and experiments for optimizing the sensitivity of curved D-type optical fiber sensor with a wide dynamic range. Opt. Commun..

[31] Sun Y.S., Zhu X.D. (2015). Kinetic Analysis of Biomolecular Interactions Using Label-Free Biosensors. Instrum. Sci. Technol..

[32] Kusnezow W., Syagailo Y.V., Ruffer S., Klenin K., Sebald W., Hoheisel J.D., Gauer C., Goychuk I. (2006). Kinetics of antigen binding to antibody microspots: Strong limitation by mass transport to the surface. Proteomics.

[33] Schuck P., Minton A.P. (1996). Analysis of mass transport-limited binding kinetics in evanescent wave biosensors. Anal. Biochem..

[34] Hsu W.T., Hsieh W.H., Cheng S.F., Jen C.P., Wu C.C., Li C.H., Lee C.Y., Li W.Y., Chau L.K., Chiang C.Y. (2011). Integration of fiber optic-particle plasmon resonance biosensor with microfluidic chip. Anal. Chim. Acta.

[35] Makiabadi T., Le Nader V., Kanso M., Louarn G. (2011). Comprehensive study of an optical fiber plasmonic microsensor in a microfluidic device. Eur. Phys. J. Appl. Phys..

[36] Wu S.Y., Hou H.S., Sun Y.S., Cheng J.Y., Lo K.Y. (2015). Correlation between cell migration and reactive oxygen species under electric field stimulation. Biomicrofluidics.

[37] Lo K.Y., Zhu Y., Tsai H.F., Sun Y.S. (2013). Effects of shear stresses and antioxidant concentrations on the production of reactive oxygen species in lung cancer cells. Biomicrofluidics.

[38] Sun Y.S., Peng S.W., Cheng J.Y. (2012). In vitro electrical-stimulated wound-healing chip for studying electric field-assisted wound-healing process. Biomicrofluidics.

[39] Sun Y.S., Peng S.W., Lin K.H., Cheng J.Y. (2012). Electrotaxis of lung cancer cells in ordered three-dimensional scaffolds. Biomicrofluidics.

[40] Esteban O., Diaz-Herrera N., Navarrete M.C., Gonzalez-Cano A. (2006). Surface plasmon resonance sensors based on uniform-waist tapered fibers in a reflective configuration. Appl. Opt..

[41] Bueno F.J., Esteban O., Diaz-Herrera N., Navarrete M.C., Gonzalez-Cano A. (2004). Sensing properties of asymmetric double-layer-covered tapered fibers. Appl. Opt..

[42] Song X.F., Wang Z.J., Liu Y.B., Wang C., Li L.J. (2009). A highly sensitive ethanol sensor based on mesoporous ZnO-SnO_2_ nanofibers. Nanotechnology.

[43] Shabaneh A.A., Girei S.H., Arasu P.T., Rashid S.A., Yunusa Z., Mahdi M.A., Paiman S., Ahmad M.Z., Yaacob M.H. (2014). Reflectance Response of Optical Fiber Coated With Carbon Nanotubes for Aqueous Ethanol Sensing. IEEE Photonics J..

[44] Morisawa M., Muto S. (2012). Plastic Optical Fiber Sensing of Alcohol Concentration in Liquors. J. Sens..

[45] Rahman M.M., Khan S.B., Jamal A., Faisal M., Asiri A.M. (2012). Fabrication of a methanol chemical sensor based on hydrothermally prepared alpha-Fe_2_O_3_ codoped SnO_2_ nanocubes. Talanta.

[46] Rahman M.M., Khan S.B., Jamal A., Faisal M., Asiri A.M. (2012). Highly sensitive methanol chemical sensor based on undoped silver oxide nanoparticles prepared by a solution method. Microchim. Acta.

[47] Fabian M., Lewis E., Newe T., Lochmann S., Mueller I. Investigation of ethanol and methanol water mixtures in the visible wavelength area using fibre-optic evanescent field absorption sensors based on a u-shaped, a coil-shaped and a meander-shaped probe. Proceeding of the SAS 2008-IEEE Sensors Applications Symposium.

[48] Wang C.J., Kaya M., Wang C. (2012). Evanescent field-fiber loop ringdown glucose sensor. J. Biomed. Opt..

[49] Luo B.B., Yan Z.J., Sun Z.Y., Liu Y., Zhao M.F., Zhang L. (2015). Biosensor based on excessively tilted fiber grating in thin-cladding optical fiber for sensitive and selective detection of low glucose concentration. Opt. Express.

[50] Li L., Walt D.R. (1995). Dual-analyte fiber-optic sensor for the simultaneous and continuous measurement of glucose and oxygen. Anal. Chem..

